# Biphenyl-2,2′,4,4′-tetra­carb­oxy­lic acid monohydrate

**DOI:** 10.1107/S1600536810037438

**Published:** 2010-09-25

**Authors:** Dong Bu, Ai-yun Zhang, Dan Zhao

**Affiliations:** a Department of Physics and Chemistry, Henan Polytechnic University, Jiaozuo 454000, People’s Republic of China

## Abstract

In the title compound, C_16_H_10_O_8_·H_2_O, the dihedral angle between the two benzene rings is 71.63 (5)°. In the crystal structure, pairs of inversion-related mol­ecules are stacked [mean inter­planar spacing = 3.5195 (18) Å], and O—H⋯O and C—H⋯O hydrogen bonds create a three-dimensional network.

## Related literature

For general background to the use of aromatic carboxyl­ates as building blocks for the construction of various architectures, see: Li *et al.* (2008 ▶); Du *et al.* (2007 ▶). For previous studies on the synthesis of aromatic carboxyl­ate hydrates, see: Jiang *et al.* (2008 ▶); Li *et al.* (2009 ▶).
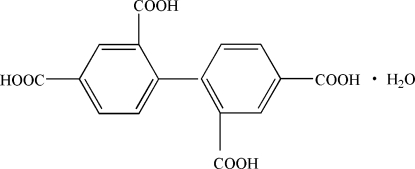


## Experimental

### 

#### Crystal data

C_16_H_10_O_8_·H_2_O
*M* *_r_* = 348.26
Triclinic, 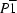

*a* = 7.1765 (1) Å
*b* = 9.4677 (2) Å
*c* = 11.9301 (2) Å
α = 106.013 (1)°
β = 100.098 (1)°
γ = 96.753 (1)°
*V* = 755.18 (2) Å^3^
*Z* = 2
Mo *K*α radiation
μ = 0.13 mm^−1^
*T* = 296 K
0.22 × 0.20 × 0.19 mm

#### Data collection

Bruker APEXII CCD area-detector diffractometer
Absorption correction: multi-scan (*SADABS*; Bruker, 2007 ▶) *T*_min_ = 0.972, *T*_max_ = 0.976
13177 measured reflections
2663 independent reflections
2441 reflections with *I* > 2σ(*I*)
*R*_int_ = 0.019

#### Refinement

*R*[*F* ^2^ > 2σ(*F* ^2^)] = 0.041
*wR*(*F* ^2^) = 0.123
*S* = 1.03
2663 reflections
236 parameters
3 restraints
H atoms treated by a mixture of independent and constrained refinement
Δρ_max_ = 0.22 e Å^−3^
Δρ_min_ = −0.41 e Å^−3^

### 

Data collection: *APEX2* (Bruker, 2007 ▶); cell refinement: *SAINT* (Bruker, 2007 ▶); data reduction: *SAINT*; program(s) used to solve structure: *SHELXS97* (Sheldrick, 2008 ▶); program(s) used to refine structure: *SHELXL97* (Sheldrick, 2008 ▶); molecular graphics: *SHELXTL* (Sheldrick, 2008 ▶); software used to prepare material for publication: *SHELXTL*.

## Supplementary Material

Crystal structure: contains datablocks I, global. DOI: 10.1107/S1600536810037438/pk2260sup1.cif

Structure factors: contains datablocks I. DOI: 10.1107/S1600536810037438/pk2260Isup2.hkl

Additional supplementary materials:  crystallographic information; 3D view; checkCIF report

## Figures and Tables

**Table 1:** Hydrogen-bond geometry (Å, °)

*D*—H⋯*A*	*D*—H	H⋯*A*	*D*⋯*A*	*D*—H⋯*A*
O6—H6*A*⋯O9	0.82	1.79	2.6076 (18)	174
O1—H1*A*⋯O2^i^	0.82	1.87	2.680 (2)	169
O3—H3*A*⋯O4^ii^	0.82	1.84	2.6400 (17)	166
O7—H7*A*⋯O5^iii^	0.82	1.82	2.6280 (17)	171
O9—H9*B*⋯O8^iv^	0.85 (1)	1.92 (1)	2.7616 (19)	170 (2)
O9—H9*A*⋯O8^v^	0.84 (1)	2.20 (1)	2.983 (2)	154 (2)
C12—H12⋯O3^vi^	0.93	2.57	3.451 (2)	159

Symmetry codes: (i) 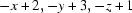; (ii) 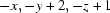; (iii) 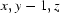; (iv) 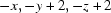; (v) 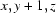; (vi) 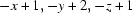.

**Fig. 1.**
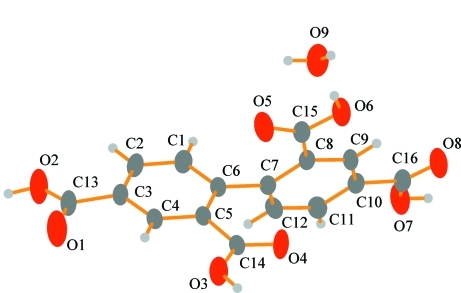
The structure of the title compound showing the atom-labeling scheme. Displacement ellipsoids are drawn at the 50% probability level

**Fig. 2.**
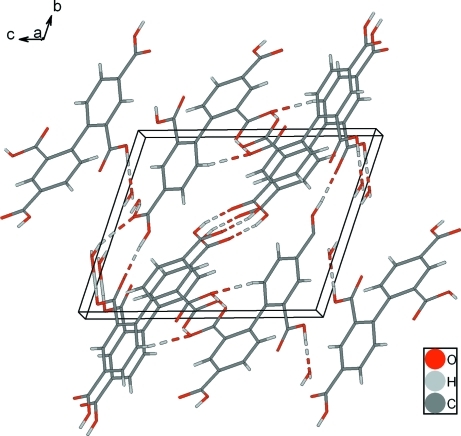
The O···H—O and C—H···O hydrogen bonds of biphenyl-2, 2', 4, 4' -tetracarboxylic acid monhydrate.

## supplementary crystallographic information

### Comment

Aromatic carboxylates have been proven to be effective building blocks in constructing various architectures (Li *et al.*, 2008; Du *et al.*, 2007). Many crystal structures of aromatic carboxylic hydrates have been reported (Jiang *et al.*, 2008; Li *et al.*, 2009). In this paper, we report the synthesis and structure of a new aromatic carboxylic hydrate, biphenyl-2, 2', 4, 4' -tetracarboxylic acid monohydrate, (Fig. 1).

The dihedral angle between the two benzene rings of biphenyl-2, 2', 4, 4' -tetracarboxylic acid monohydrate is 71.63 (5)°, which is markedly different from 42.30 (11)° found in the biphenyl-2, 3, 3', 4'-tetracarboxylic acid monohydrate (Jiang *et al.*, 2008).  This might be a result of the hydrogen bonding ineractions of the title compound.  The lattice water molecule links with biphenyl-2, 2', 4, 4'-tetracarboxylic acid *via* O—H···O hydrogen bonding.  The extensive O—H···O hydrogen bonding and a weak intermolecular C—H···O hydrogen bond helps to stabilize the crystal structure (Fig. 2 and Table 1).  In addition, pairs of inversion related molecules are stacked with mean interplanar spacing = 3.5195 (18)Å)

### Experimental

A mixture of C_16_H_10_O_8_ (0.3360 g), BaCl_2_ (0.3451 g) and water (12 ml) was stirred at room temperature for 6 h. The solution was filtered and the filtrate was left to stand undisturbed.  Upon slow evaporation at room temperature, a colorless crystalline solid appeared about a month later. The resulting colorless blocks were filtered off washed with water and dried at ambient temperature.

### Refinement

The H atoms of water molecules were located in difference Fourier maps and refined.  All other hydrogen atoms were included in calculated positions and refined using a riding model with isotropic thermal parameters derived from the parent atoms (C—H = 0.93Å, O—H = 0.82 Å, *U*_iso_ (H) = 1.2Ueq (C) or 1.5Ueq (O)).

### Crystal data

**Table:**

C_16_H_10_O_8_·H_2_O	*Z* = 2
*M**_r_* = 348.26	*F*(000) = 360
Triclinic, *P*1	*D*_x_ = 1.532 Mg m^−^^3^
Hall symbol: -P 1	Mo *K*α radiation, λ = 0.71073 Å
*a* = 7.1765 (1) Å	Cell parameters from 9316 reflections
*b* = 9.4677 (2) Å	θ = 2.8–27.5°
*c* = 11.9301 (2) Å	µ = 0.13 mm^−^^1^
α = 106.013 (1)°	*T* = 296 K
β = 100.098 (1)°	Block, yellow
γ = 96.753 (1)°	0.22 × 0.20 × 0.19 mm
*V* = 755.18 (2) Å^3^	

### Data collection

**Table:**

Bruker APEXII CCD area-detector diffractometer	2663 independent reflections
Radiation source: fine-focus sealed tube	2441 reflections with *I* > 2σ(*I*)
graphite	*R*_int_ = 0.019
φ and ω scans	θ_max_ = 25.0°, θ_min_ = 1.8°
Absorption correction: multi-scan (*SADABS*; Bruker, 2007)	*h* = −8→8
*T*_min_ = 0.972, *T*_max_ = 0.976	*k* = −11→11
13177 measured reflections	*l* = −14→14

### Refinement

**Table:**

Refinement on *F*^2^	Primary atom site location: structure-invariant direct methods
Least-squares matrix: full	Secondary atom site location: difference Fourier map
*R*[*F*^2^ > 2σ(*F*^2^)] = 0.041	Hydrogen site location: inferred from neighbouring sites
*wR*(*F*^2^) = 0.123	H atoms treated by a mixture of independent and constrained refinement
*S* = 1.03	*w* = 1/[σ^2^(*F*_o_^2^) + (0.0701*P*)^2^ + 0.3599*P*] where *P* = (*F*_o_^2^ + 2*F*_c_^2^)/3
2663 reflections	(Δ/σ)_max_ < 0.001
236 parameters	Δρ_max_ = 0.22 e Å^−^^3^
3 restraints	Δρ_min_ = −0.40 e Å^−^^3^

### Special details

**Table:**

Geometry. All e.s.d.'s (except the e.s.d. in the dihedral angle between two l.s. planes) are estimated using the full covariance matrix. The cell e.s.d.'s are taken into account individually in the estimation of e.s.d.'s in distances, angles and torsion angles; correlations between e.s.d.'s in cell parameters are only used when they are defined by crystal symmetry. An approximate (isotropic) treatment of cell e.s.d.'s is used for estimating e.s.d.'s involving l.s. planes.
Refinement. Refinement of *F*^2^ against ALL reflections. The weighted *R*-factor *wR* and goodness of fit *S* are based on *F*^2^, conventional *R*-factors *R* are based on *F*, with *F* set to zero for negative *F*^2^. The threshold expression of *F*^2^ > σ(*F*^2^) is used only for calculating *R*-factors(gt) *etc*. and is not relevant to the choice of reflections for refinement. *R*-factors based on *F*^2^ are statistically about twice as large as those based on *F*, and *R*- factors based on ALL data will be even larger.

### Fractional atomic coordinates and isotropic or equivalent isotropic displacement parameters (Å^2^)

**Table:**

	*x*	*y*	*z*	*U*_iso_*/*U*_eq_	
O1	0.7651 (3)	1.4462 (2)	0.51405 (19)	0.0815 (6)	
H1A	0.8362	1.4851	0.4799	0.122*	
O2	1.0447 (2)	1.4309 (2)	0.61981 (17)	0.0734 (5)	
O3	0.19262 (18)	1.12724 (18)	0.49778 (13)	0.0522 (4)	
H3A	0.0770	1.0941	0.4759	0.078*	
O4	0.17196 (18)	0.97698 (17)	0.60878 (13)	0.0518 (4)	
O5	0.3535 (2)	1.20930 (14)	0.88045 (14)	0.0531 (4)	
O6	0.1817 (2)	1.07466 (14)	0.96298 (13)	0.0448 (3)	
H6A	0.1502	1.1551	0.9916	0.067*	
O7	0.4508 (2)	0.46084 (14)	0.83504 (15)	0.0531 (4)	
H7A	0.4132	0.3873	0.8539	0.080*	
O8	0.2313 (2)	0.55361 (14)	0.92899 (13)	0.0474 (4)	
O9	0.1086 (2)	1.34013 (16)	1.05603 (16)	0.0556 (4)	
C1	0.7674 (2)	1.1428 (2)	0.77090 (16)	0.0376 (4)	
H1	0.8355	1.1113	0.8306	0.045*	
C2	0.8611 (3)	1.2492 (2)	0.73154 (17)	0.0398 (4)	
H2	0.9908	1.2877	0.7640	0.048*	
C3	0.7614 (2)	1.29848 (19)	0.64325 (15)	0.0341 (4)	
C4	0.5674 (2)	1.24238 (18)	0.59864 (14)	0.0308 (4)	
H4	0.4990	1.2783	0.5419	0.037*	
C5	0.4723 (2)	1.13359 (17)	0.63672 (13)	0.0273 (3)	
C6	0.5744 (2)	1.08125 (17)	0.72423 (14)	0.0283 (4)	
C7	0.4997 (2)	0.95149 (17)	0.76245 (14)	0.0278 (3)	
C8	0.3751 (2)	0.95295 (17)	0.84121 (14)	0.0275 (3)	
C9	0.3250 (2)	0.82523 (17)	0.87296 (14)	0.0288 (4)	
H9	0.2409	0.8259	0.9241	0.035*	
C10	0.3984 (2)	0.69691 (17)	0.82967 (14)	0.0298 (4)	
C11	0.5231 (3)	0.69593 (18)	0.75326 (15)	0.0346 (4)	
H11	0.5729	0.6103	0.7236	0.041*	
C12	0.5734 (2)	0.82211 (19)	0.72119 (15)	0.0337 (4)	
H12	0.6588	0.8206	0.6707	0.040*	
C13	0.8643 (3)	1.4007 (2)	0.59059 (18)	0.0441 (5)	
C14	0.2659 (2)	1.07440 (18)	0.57925 (14)	0.0289 (4)	
C15	0.3025 (2)	1.09080 (18)	0.89507 (15)	0.0305 (4)	
C16	0.3493 (2)	0.56452 (18)	0.86928 (15)	0.0326 (4)	
H9B	0.0046 (19)	1.370 (2)	1.0685 (19)	0.049*	
H9A	0.177 (2)	1.4042 (19)	1.037 (2)	0.049*	

### Atomic displacement parameters (Å^2^)

**Table:**

	*U*^11^	*U*^22^	*U*^33^	*U*^12^	*U*^13^	*U*^23^
O1	0.0562 (10)	0.1074 (15)	0.0975 (14)	−0.0142 (10)	0.0034 (9)	0.0776 (12)
O2	0.0451 (9)	0.0946 (13)	0.0903 (13)	−0.0128 (8)	0.0080 (8)	0.0581 (11)
O3	0.0305 (7)	0.0725 (10)	0.0609 (9)	−0.0063 (6)	−0.0048 (6)	0.0472 (8)
O4	0.0309 (7)	0.0645 (9)	0.0665 (9)	−0.0116 (6)	−0.0020 (6)	0.0463 (8)
O5	0.0791 (10)	0.0269 (7)	0.0717 (10)	0.0150 (6)	0.0410 (8)	0.0268 (6)
O6	0.0483 (8)	0.0325 (7)	0.0667 (9)	0.0130 (6)	0.0330 (7)	0.0210 (6)
O7	0.0648 (9)	0.0334 (7)	0.0836 (10)	0.0199 (7)	0.0408 (8)	0.0341 (7)
O8	0.0514 (8)	0.0381 (7)	0.0709 (9)	0.0136 (6)	0.0310 (7)	0.0326 (7)
O9	0.0501 (9)	0.0410 (8)	0.0905 (11)	0.0181 (6)	0.0342 (8)	0.0280 (8)
C1	0.0303 (9)	0.0426 (10)	0.0421 (9)	−0.0012 (7)	0.0001 (7)	0.0243 (8)
C2	0.0291 (9)	0.0432 (10)	0.0462 (10)	−0.0070 (7)	0.0026 (7)	0.0210 (8)
C3	0.0327 (9)	0.0336 (9)	0.0372 (9)	−0.0034 (7)	0.0087 (7)	0.0158 (7)
C4	0.0323 (9)	0.0319 (8)	0.0319 (8)	0.0013 (7)	0.0071 (7)	0.0172 (7)
C5	0.0268 (8)	0.0283 (8)	0.0293 (8)	0.0022 (6)	0.0083 (6)	0.0125 (6)
C6	0.0290 (8)	0.0278 (8)	0.0306 (8)	0.0020 (6)	0.0081 (6)	0.0130 (6)
C7	0.0270 (8)	0.0286 (8)	0.0292 (8)	0.0011 (6)	0.0027 (6)	0.0147 (6)
C8	0.0264 (8)	0.0256 (8)	0.0328 (8)	0.0024 (6)	0.0051 (6)	0.0144 (6)
C9	0.0288 (8)	0.0274 (8)	0.0350 (8)	0.0029 (6)	0.0103 (6)	0.0156 (6)
C10	0.0315 (8)	0.0252 (8)	0.0349 (8)	0.0028 (6)	0.0063 (7)	0.0142 (6)
C11	0.0409 (9)	0.0277 (8)	0.0401 (9)	0.0095 (7)	0.0136 (7)	0.0138 (7)
C12	0.0378 (9)	0.0340 (9)	0.0356 (9)	0.0068 (7)	0.0148 (7)	0.0164 (7)
C13	0.0344 (10)	0.0508 (11)	0.0503 (11)	−0.0077 (8)	0.0054 (8)	0.0289 (9)
C14	0.0274 (8)	0.0321 (8)	0.0310 (8)	0.0028 (6)	0.0075 (6)	0.0159 (6)
C15	0.0309 (8)	0.0277 (8)	0.0371 (8)	0.0049 (6)	0.0078 (7)	0.0165 (7)
C16	0.0337 (9)	0.0264 (8)	0.0422 (9)	0.0048 (6)	0.0097 (7)	0.0170 (7)

### Geometric parameters (Å, °)

**Table:**

O1—C13	1.260 (2)	C3—C4	1.384 (2)
O1—H1A	0.8200	C3—C13	1.486 (2)
O2—C13	1.256 (2)	C4—C5	1.391 (2)
O3—C14	1.274 (2)	C4—H4	0.9300
O3—H3A	0.8200	C5—C6	1.405 (2)
O4—C14	1.243 (2)	C5—C14	1.489 (2)
O5—C15	1.207 (2)	C6—C7	1.498 (2)
O6—C15	1.309 (2)	C7—C12	1.391 (2)
O6—H6A	0.8200	C7—C8	1.405 (2)
O7—C16	1.309 (2)	C8—C9	1.390 (2)
O7—H7A	0.8200	C8—C15	1.484 (2)
O8—C16	1.211 (2)	C9—C10	1.385 (2)
O9—H9B	0.852 (9)	C9—H9	0.9300
O9—H9A	0.843 (9)	C10—C11	1.384 (2)
C1—C2	1.378 (2)	C10—C16	1.483 (2)
C1—C6	1.389 (2)	C11—C12	1.380 (2)
C1—H1	0.9300	C11—H11	0.9300
C2—C3	1.387 (3)	C12—H12	0.9300
C2—H2	0.9300		
			
C13—O1—H1A	109.5	C9—C8—C15	119.43 (14)
C14—O3—H3A	109.5	C7—C8—C15	120.93 (14)
C15—O6—H6A	109.5	C10—C9—C8	121.11 (15)
C16—O7—H7A	109.5	C10—C9—H9	119.4
H9B—O9—H9A	109.6 (14)	C8—C9—H9	119.4
C2—C1—C6	122.08 (16)	C11—C10—C9	119.36 (14)
C2—C1—H1	119.0	C11—C10—C16	120.69 (15)
C6—C1—H1	119.0	C9—C10—C16	119.91 (15)
C1—C2—C3	119.76 (16)	C12—C11—C10	119.95 (15)
C1—C2—H2	120.1	C12—C11—H11	120.0
C3—C2—H2	120.1	C10—C11—H11	120.0
C4—C3—C2	119.01 (15)	C11—C12—C7	121.64 (15)
C4—C3—C13	120.32 (16)	C11—C12—H12	119.2
C2—C3—C13	120.49 (16)	C7—C12—H12	119.2
C3—C4—C5	121.54 (15)	O2—C13—O1	123.66 (18)
C3—C4—H4	119.2	O2—C13—C3	118.64 (17)
C5—C4—H4	119.2	O1—C13—C3	117.59 (16)
C4—C5—C6	119.40 (14)	O4—C14—O3	122.26 (15)
C4—C5—C14	117.74 (14)	O4—C14—C5	121.23 (14)
C6—C5—C14	122.84 (14)	O3—C14—C5	116.51 (14)
C1—C6—C5	118.14 (14)	O5—C15—O6	122.33 (16)
C1—C6—C7	115.96 (14)	O5—C15—C8	123.22 (15)
C5—C6—C7	125.51 (14)	O6—C15—C8	114.42 (14)
C12—C7—C8	118.34 (14)	O8—C16—O7	123.12 (15)
C12—C7—C6	115.43 (14)	O8—C16—C10	124.19 (15)
C8—C7—C6	126.07 (14)	O7—C16—C10	112.68 (14)
C9—C8—C7	119.59 (15)		
			
C6—C1—C2—C3	0.5 (3)	C8—C9—C10—C11	−0.2 (2)
C1—C2—C3—C4	1.7 (3)	C8—C9—C10—C16	177.30 (15)
C1—C2—C3—C13	−173.46 (18)	C9—C10—C11—C12	0.2 (3)
C2—C3—C4—C5	−2.7 (3)	C16—C10—C11—C12	−177.33 (16)
C13—C3—C4—C5	172.56 (16)	C10—C11—C12—C7	−0.9 (3)
C3—C4—C5—C6	1.3 (2)	C8—C7—C12—C11	1.7 (2)
C3—C4—C5—C14	−176.96 (15)	C6—C7—C12—C11	177.43 (15)
C2—C1—C6—C5	−1.9 (3)	C4—C3—C13—O2	−168.9 (2)
C2—C1—C6—C7	171.29 (17)	C2—C3—C13—O2	6.2 (3)
C4—C5—C6—C1	1.0 (2)	C4—C3—C13—O1	7.5 (3)
C14—C5—C6—C1	179.14 (15)	C2—C3—C13—O1	−177.4 (2)
C4—C5—C6—C7	−171.49 (15)	C4—C5—C14—O4	179.96 (16)
C14—C5—C6—C7	6.6 (2)	C6—C5—C14—O4	1.8 (3)
C1—C6—C7—C12	−67.3 (2)	C4—C5—C14—O3	0.9 (2)
C5—C6—C7—C12	105.40 (19)	C6—C5—C14—O3	−177.24 (16)
C1—C6—C7—C8	108.06 (19)	C9—C8—C15—O5	173.05 (17)
C5—C6—C7—C8	−79.3 (2)	C7—C8—C15—O5	−4.3 (3)
C12—C7—C8—C9	−1.7 (2)	C9—C8—C15—O6	−5.2 (2)
C6—C7—C8—C9	−176.93 (15)	C7—C8—C15—O6	177.43 (15)
C12—C7—C8—C15	175.65 (15)	C11—C10—C16—O8	−174.54 (17)
C6—C7—C8—C15	0.5 (2)	C9—C10—C16—O8	8.0 (3)
C7—C8—C9—C10	1.0 (2)	C11—C10—C16—O7	6.9 (2)
C15—C8—C9—C10	−176.42 (14)	C9—C10—C16—O7	−170.53 (16)

### Hydrogen-bond geometry (Å, °)

**Table:**

*D*—H···*A*	*D*—H	H···*A*	*D*···*A*	*D*—H···*A*
O6—H6A···O9	0.82	1.79	2.6076 (18)	174
C4—H4···O3	0.93	2.37	2.706 (2)	101
C9—H9···O6	0.93	2.38	2.711 (2)	101
O1—H1A···O2^i^	0.82	1.87	2.680 (2)	169
O3—H3A···O4^ii^	0.82	1.84	2.6400 (17)	166
O7—H7A···O5^iii^	0.82	1.82	2.6280 (17)	171
O9—H9B···O8^iv^	0.85 (1)	1.92 (1)	2.7616 (19)	170 (2)
O9—H9A···O8^v^	0.84 (1)	2.20 (1)	2.983 (2)	154 (2)
C12—H12···O3^vi^	0.93	2.57	3.451 (2)	159

Symmetry codes: (i) −*x*+2, −*y*+3, −*z*+1; (ii) −*x*, −*y*+2, −*z*+1; (iii) *x*, *y*−1, *z*; (iv) −*x*, −*y*+2, −*z*+2; (v) *x*, *y*+1, *z*; (vi) −*x*+1, −*y*+2, −*z*+1.

## References

[bb1] Bruker (2007). *APEX2*, *SAINT* and *SADABS* Bruker AXS Inc., Madison, Wisconsin, USA.

[bb2] Du, M., Li, C.-P., Zhao, X.-J. & Yu, Q. (2007). *CrystEngComm*, **9**, 1011–1028.

[bb3] Jiang, Y., Men, J., Liu, C.-Y., Zhang, Y. & Gao, G.-W. (2008). *Acta Cryst.* E**64**, o846. 10.1107/S1600536808009689 PMC2961247 21202334

[bb4] Li, C.-P., Tian, Y.-L. & Guo, Y.-M. (2008). *Inorg. Chem. Commun.* **11**, 1405– 1408.

[bb5] Li, F., Wang, W.-W., Ji, X., Cao, C.-C. & Zhu, D.-Y. (2009). *Acta Cryst.* E**65**, o244. 10.1107/S1600536808044012 PMC2968332 21581861

[bb6] Sheldrick, G. M. (2008). *Acta Cryst.* A**64**, 112–122. 10.1107/S0108767307043930 18156677

